# Optimization of methods for peripheral blood mononuclear cells isolation and expansion of human gamma delta T cells

**DOI:** 10.6026/97320630017460

**Published:** 2021-03-31

**Authors:** Mohd Wajid Ali Khan, Ahmed Al Otaibi, Subuhi Sherwani, Eida Mohammed Alshammari, Salma Ahmed Al-Zahrani, Wahid Ali Khan, Abdulmohsen Khalaf Dhahi Alsukaibi, Sultan Alouffi, Shahper Nazeer Khan

**Affiliations:** 1 Department of Chemistry, College of Sciences, University of Ha'il, Ha'il-2440, Saudi Arabia; 2 Molecular Diagnostic and Personalised Therapeutics Unit, University of Ha'il, Ha'il-2440, Saudi Arabia; 3 Department of Biology, College of Sciences, University of Ha'il, Ha'il-2440, Saudi Arabia; 4 Department of Clinical Biochemistry,College of Medicine, King Khalid University, Abha-62529, Saudi Arabia; 5 Department of Clinical Laboratory Sciences, College of Applied Medical Sciences, University of Ha'il, Ha'il-2440, Saudi Arabia; 6 Interdisciplinary Nanotechnology Centre, Aligarh Muslim University, Aligarh-202002, U.P,India

**Keywords:** γδT cell, expanded, cytokines, zoledronate, cancer therapeutics, PBMCs

## Abstract

Human Vg9/Vδ2 T cells (γδ T cells) are immune surveillance cells both in innate and adaptive immunity and are a possible target for anticancer therapies, which can induce immune responses in a variety of cancers. Small non-peptide antigens such as zoledronate can do activation and expansion of T cells in vitro. It is evident that for adoptive cancer therapies, large numbers of functional cells are needed into cancer patients. Hence, optimization of methods needs to be carried out for the efficient expansion of these T cells. Standardization of peripheral blood mononuclear cells (PBMCs) isolation was devised. Cytokines (interleukin 2 (IL-2) and interleukin 15 (IL-15)) and zoledronate were also standardized for different concentrations. It was found that an increased number of PBMCs were recovered when washing was done at 1100 revolution per minute (rpm). Significantly high expansion fold was (2524 ± 787 expansion fold) achieved when stimulation of PBMCs was done with 1 µM of zoledronate and both cytokines IL-2 and IL-15 supported the expansion and survival of cells at the concentrations of 100 IU/ml and 10 ng/ml respectively. 14-day cultures showed highly pure (91.6 ± 5.1%) and live (96.5 ± 2.5%) expanded γδ T cells. This study aimed to standardize an easy to manipulate technique for the expansion of γδ T cells, giving a higher yield.

## Background

Cancer is a disease distinguished by abnormal growth of cells, which leads to morbidity and mortality worldwide, with approximately 18.1 million new cases and 9.6 million cancer deaths according to the International Agency for Research on Cancer in 2018 [1]. It is estimated that in 2020, 1.8 million new cancer cases would be diagnosed and about 606,520 cancer-related mortalities are predicted in the US [2]. Immune cells play an integral role in tumour cell control (immunosurveillance), and immune defects are frequently associated with cancer development and disease progression [3]. Consequently, corrective measures aimed at restoring anti-tumour immunity are a major focus in current research to develop novel cancer immunotherapies. T-cell based cancer immunotherapy can be carried out using in vitro expanded and differentiated effectors cells. The activated immune cells are transferred back to the patients as personalized cellular vaccines. These cells either target tumor cells directly or eliminate them by stimulating the immune response [4]. Over the last decade, human Vg9/Vδ2 T cells (γδ T cells) have become an ideal effector cell option for cancer immunotherapy. The two main types of T lymphocytes found in human peripheral blood are αβ and gδ T cells. These Vg9/Vδ2 cells express a T cell receptor (TCR) comprising of γ and δ chains, that are unique to humans and higher primates [5]. γδ T cells are atypical T cells that can recognize and lyse diverse cancer cells in a non- major histocompatibility complex (MHC) restricted manner, highlighting their potential for cancer immunotherapy. They vigorously contribute to the anti-tumor immune response in many cancers (colon, breast, lymphoma, myeloma, melanoma, lungs, ovary and prostate) [6]. γδ T cells perform these activities through cytotoxicity against tumor/infected cells like CD8+ T cells/NK cells and by regulating other immune cells (antigen presentation function) such as dendritic cells, macrophages and B cells [7-9]. Human PBMCs usually contain 1-10% of circulating γδ T cells [5]. gδ T cells offer a selective and non-MHC restricted response to phosphoantigens and, indirectly, to aminobisphosphonates [5]. They also behave as antigen presenting cells (APCs) [10]. The γδ cells and are excellent at cross-presentation of protein antigens, which is relevant for CD8+ T cell driven anti-tumour immunity [10]. Wada et al. [11] used ex vivo-expanded γδ T-cells for the treatment of gastric cancer linked malignant ascites in the peritoneum. Injection of γδ T-cells into the peritoneal cavity allow effective access to tumor cells. Computed tomography revealed significant reduction in the ascite volume in two patients out of seven, with most commonly observed treatment related side effects as mild fever and zoledronate-induced hypocalcemia [11]. γδ T cells have the potential to be used as cancer therapeutics. Large numbers of such expanded cells are infused back into the cancer patients. In this study, we standardized and established a protocol for the proliferation of large numbers of γδ T cells with high purity, using minimum amounts of human blood.

## Methodology

### Chemicals and reagents:

RPMI-1640 medium, LymphoprepTM, 0.9% saline (NaCl), Falcon tube (50 ml), Millipore filter (0.22 µM), syringe, 10% Foetus calf serum (FCS), L-glutamine (200mM; 100X), Pen/strep (10,000 units/ml pen and 10,000 units/ml strep), MEM Sodium pyruvate (100mM), non-essential amino acids (10mM; 100X). IL-2 (100 IU/ml) were from GIBCO Life Technologies (USA). Interleukin-2 and 15 were purchased from Miltenyi Biotec (Germany). Monoclonal antibodies (mAbs) specific for CD3, CD4, CD8, TCR-Vγ9 were from Beckman Coulter (USA). Live/Dead fixable aqua dead cell stain kit was from Invitrogen-Life Technologies (USA). Immunoglobulin from human and zoledronic acid injection (4 mg) were purchased from Sigma Aldrich and Cipla respectively.

### Collection of fresh blood samples:

Venous blood samples were collected from healthy individuals (males aged 25-45 years) with their full consent. This study was performed in accordance with the ethical standards as given in the Helsinki Declaration of 1975 and revised in 2000. Ethical approval for this study was received from the institution. The following procedure was adopted for venous blood collection; a proper sized needle was attached to the syringe. For best visualization of the draw site was positioned and tourniquet ideally applied 3-4 inches above the puncture site. The participants were instructed to make a fist and hold it without pumping their hand. The venipuncture site was prepared using a Chlorhexadine wipe followed by air-drying. The needle was inserted into the skin, at an angle of 15-30 degree to the skin, into the vein. All the standard safety measures were followed while drawing blood.

### Preparation of RPMI medium:

An unopened RPMI-1650 (500 ml) was opened under a laminar flow in a sterile environment and 70 ml of medium was removed. 50 ml (10%) FCS (heat inactivated), 5 ml L-glutamine (200mM; 100X), 5 ml Pen/strep, 5 ml MEM Sodium Pyruvate, and 5 ml MEM non-essential amino acids were added to this. To avoid contamination, all solutions were passed through Millipore filters (0.22 µM) before being added to RPMI medium.

### Peripheral blood mononuclear cells Isolation:

A volume of 5 ml fresh blood was drawn using the method stated above. A 50 ml sterile falcon tube was taken to which 5 ml heparin/EDTA was added. Blood from the syringe was transferred to this tube and tilting the tube sideways facilitated mixing with EDTA. Five ml lymphoprepTM was added to a sterile 15 ml falcon tube and brought to room temperature. Blood was added to the lymphoprepTM, by tilting the tube and placing the tip of the pipette on the surface of the upper layer while pouring gently. Two layers separated out: blood in the upper layer and lymphoprepTM at the bottom. The tube was centrifuged at 1680 rpm for 22 min at 18°C with acceleration set at 2 and deceleration at 0. After centrifugation, four layers were visible; the uppermost was plasma, the second hazy thin layer contained PBMCs, third layer was lymphoprepTM and bottom most layer contained RBCs. Plasma was removed by pipetting and only a few ml (2-3 ml) was left in the tube so as not to disturb any leftover PBMCs. 10 ml sterile and cold saline (0.9% NaCl) was taken in a sterile 15 ml falcon tube. The remaining plasma and the hazy layer on top of the lymphoprepTM contained PBMCs. Approximately 3-4 ml solution was collected and added to a 15 ml tube with 10 ml normal saline and centrifuged at 1100 rpm for 10 min at 4°C. After centrifugation, the supernatant was removed, first by pipetting and later using 3 ml dropper so that the supernatant could be decanted carefully, and the pellet remained undisturbed. 1 ml saline was added to the pellet and mixed well. The tip of the pipette remained inside the solution to avoid any bubbles while mixing. A further 9 ml saline was added, and the solution centrifuged at 1100 rpm for 10 min at 4°C. After centrifugation, the supernatant was removed. 1 ml saline was added again to the pellet, mixed well and an additional 9 mL saline was also added. To estimate the number of PBMCs per mL at this stage, 20 µL of the sample was removed and the cells counted with the help of a Neubauer chamber, under a microscope (X Í 10,000 = Y million/ml). PBMC numbers usually ranged between 1-2 million cells/mL bloods. The solution was centrifuged again at 1100 rpm for 10 min at 18°C. After centrifugation, the supernatant was removed, and the pellet retained. 1 ml complete RPMI (pre-warmed) medium was added to the pellet and mixed well. The remaining volume required for an experiment was made up with complete RPMI medium. For the experiment described below 9 ml RPMI medium was added to the cells to make up the volume up to 10 ml, which contained 1 million/ml PBMCs.

### In vitro expansion of γδT cells:

γδT cell in isolated PBMCs were stimulated using 1µM zoledronate and were cultured in complete RPMI 1640 medium at a density of 106 cells/ml in 24-well plate and kept in CO2 (5%) incubator at 37°C. The level of water in the incubator was checked often to prevent decrease in levels. Cytokines IL-2 (100 IU/ml) and IL-15 (20 ng/ml) were added to the culture at day 3, 6, 8 and 11 and cells were split into fresh medium. At day 14, the percent γδT cells in culture were evaluated by flow cytometry together with the viability. Expansion fold was also determined.

### Cell surface staining:

Approximately 50,000-500,000 PBMCs or expanded were plated in a total volume of up to 250 µl. These were centrifuge at 1100 rpm for 3 min at 4°C in a centrifuge. The pellets of cells were then washed with 200 µl phosphate buffer saline (PBS). Three microliters of Live/Dead fixable aqua dead cell stain were added and incubated for 15 min in the dark followed by the addition of 200 µl PBS contains 2% FCS (FACS buffer). Post incubation, cells were centrifuged (1100 rpm for 3 mins) and the supernatant discarded. Cells were blocked with 100 µl of 1:1000 diluted human IgG for 15 min on ice. After incubation cells were centrifuged (1100 rpm for 3 mins) and throw the supernatant. Then stained cells with 40 µl cocktails of antibodies and isotype by further incubating for 20 min on ice in the dark. Approximately 160 µl FACS buffer was added after incubation and then the mixture was centrifuged (1100 rpm for 3 mins). Finally, cells were resuspended in 100 µl FACS buffer and are ready for analysis at flowcytometer (Beckman Coulter Flowcytometer, Navios A52101).

## Results:

### Optimization of PBMCs Isolation from fresh blood:

Proliferated γδ T cell numbers depend on the starting number of cells. For this reason, the PBMC washing procedure during isolation from human blood was standardized to recover as many cells as possible. For the optimization process, PBMCs were centrifuged at different rpm (1400, 1300, 1100, 900, 800 and 600) (Table 1). Significantly higher numbers of PBMCs were recovered when washings of PBMCs was done at 1100 rpm as compared to other rpms (1400, 1300, 900, 800 and 600). After comparing the centrifugation speeds used during the washing procedure of PBMCs it was evident that 1100 rpm is an optimal speed to be used for future washing steps.

Similarly, different IL-15 concentrations (1-100 ng/ml) were used in γδ T cell cultures and plates. Different concentrations of IL-15 were added at day 3, 5, 8 and 11. Significantly high percent of live cells (P < 0.001) and γδ T cells (P < 0.001) were observed at 10 ng/ml and 100 ng/ml (Figure 2) compared to 1 ng/ml IL-2 concentration. However, at 1 ng/ml of IL-15, both live cells and γδ T cells were found to be remarkably low after 14 days culture.

### Optimization of cytokines IL-2 and IL-15:

**Figure 1:**
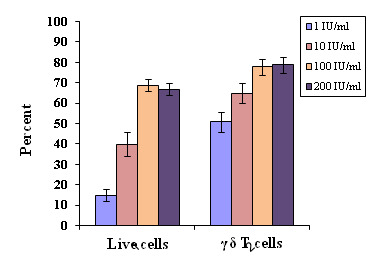
Optimization of cytokine IL-2 for the optimal proliferation of γδT cells at constant concentration of zoledronate (1 µM). Varying concentrations of IL-2 (1, 10, 100 and 200 IU/ml) were used in culture plates. Experiments were done in triplicates. For live cells, significances are defined as P<0.001 and P<0.01, when compared between the concentrations of IL-2, 100 IU/ml and 1 IU/ml, and between concentrations 10 IU/ml and 1 IU/ml respectively. For γδT cells, significance is defined as P < 0.01, when compared between the concentrations of IL-2, 100 IU/ml and 1 IU/ml, and between 10 IU/ml and 1 IU/ml.

**Figure 2:**
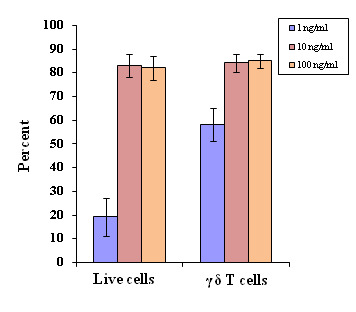
Optimization of cytokine IL-15 for the optimal proliferation of γδT cells at constant concentration of zoledronate (1 µM). Varying concentrations of IL-15 (1, 10 and 100 ng/ml) were used in culture plates under similar experimental conditions. Experiments were done in triplicates. For live cells, significance is defined as P<0.001, when compared between the concentrations of IL-15, 100 ng/ml and 1 ng/ml and, between 10 ng/ml and 1 ng/ml. For γδT cells, significance is defined as P < 0.01, when compared between the concentrations of IL-15, 100 ng/ml and 1 ng/ml and between 10 ng/ml and 1 ng/ml.

Both IL-2 and IL-15 were standardized to determine optimal concentrations to be used for γδ T proliferation. Different concentrations of IL-2 (1-200 IU/ml) were added to γδ T cell culture plates at day 3, 5, 8 and 11, respectively. Significant differences were observed in live cells, when expanded with different concentrations of IL-2. A significantly high percent of live cells was achieved with both IL-2 100 and 200 IU/ml when compared with 1 IU/ml (p < 0.001) and 10 IU/ml (p < 0.01). Furthermore, high percent of γδ T cells (P < 0.01) were observed at 100 IU/ml of IL-2 as compared to 1 and 10 IU/ml (Figure 1). Moreover, similar results were observed with 200 IU/ml of IL-2. Therefore, our protocols for γδ T cell expansion include a combination of both IL-2 and IL-15 at the concentrations of 100 IU/ml and 10 ng/ml, respectively. These concentrations were used in the optimization of zoledronate, which is a well-known stimulant of γδ T cells.

### Standardization of zoledronate:

**Figure 3:**
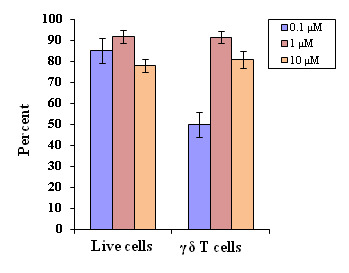
Standardization of zoledronate for the expansion of γδT cells at constant concentration of cytokines (IL-2 (100 IU/ml) and IL-15 (20 ng/ml). Different concentrations of zoledronate (0.1, 1 and 10 µM) were used in culture plates under similar experimental conditions. Experiments were done in triplicates. For γδT cells, significance is defined as P < 0.001 and P < 0.01, when compared between the concentrations of zoledronate 1 µM and 0.1 µM, and between 10 µM and 0.1 µM, respectively.

Different concentrations of zoledronate (0.1, 1, 10 µM), used as stimulant for γδ T cells, were analyzed for cell expansion in presence of both cytokines, IL-2 and IL-15. The concentration of 1 uM zoledronate led to increased percent of live expanded cells with significantly high percent of γδ T cells when compared to the 0.1 µM (p < 0.001). Furthermore, there was a significant high (p < 0.01) percent of γδ T cells were observed at 10 µM concentrations of zoledronate in 14 day cultures (Figure 3).

### Expansion folds for 14-day cultures:

**Figure 4:**
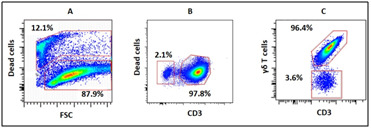
Flowcytometric analysis of 14 day expanded γδ T cells. Figure A, B and showed percent live cells, CD3 cells and γδ T cells, respectively. These data are from one donor.

In vitro proliferation of 1 µM zoledronate stimulated γδ T cells for 14 days was carried out using different cytokine(s); first condition IL-2 (100 IU/ml) alone; second condition IL-15 (10 ng/ml) alone; and third condition with both IL-2 (100 IU/ml) and IL-15 (10 ng/ml) together (Table 2). PBMCs were isolated from fresh blood from five healthy donors and expansion folds were calculated for all three-culture conditions. An expansion fold of 2524 ± 787 was estimated for γδ T cells with both cytokines (IL-2 and IL-15), which was remarkably higher (p < 0.0001) than the expansion folds calculated for the two other γδ T cell culture conditions i.e., using cytokine IL-2 alone (expansion fold 318 ± 121) or IL-15 (expansion fold 400 ± 134) alone (Table 2). Dot plots (Figure 4) show representation of percent live cells, CD3 and γδ T cells after 14 days of expansion in presence of IL-2 and IL-15. Remarkable number of live cells (87.9%) can be observed in Figure 4A, together with high percent of CD3 (97.8%) (Figure 4B) and γδ T cells (96.4%) (Figure 4C).

**Figure 5:**
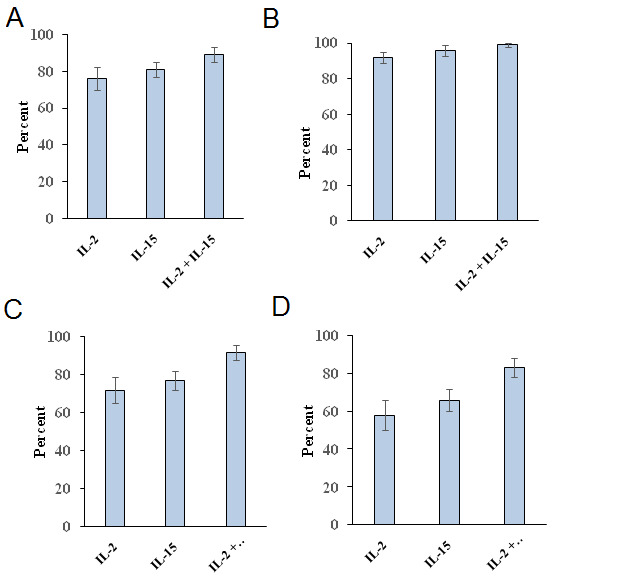
Expression of cell surface activation markers CD 69 (A), HLA-DR (B) and costimulatory markers CD80 (C) and CD86 (D) on 14 day expanded γδ T cells in presence of IL-2 or IL-15 alone or in combination of both IL-2 and IL-15. These data were from 5 donors. Experiments were done in triplicates. For CD69, significance is defined as P < 0.05 when compared with the combination of IL2 and IL-15 and IL-2 alone or IL-15 alone. For CD80, significance is defined as P < 0.05 when compared with the combination of IL2 and IL-15 and IL-2 alone or IL-15 alone. For CD86, significance is defined as P < 0.01 when compared with the combination of IL2 and IL-15 and IL-2 alone or IL-15 alone.

These expanded γδ T cells were also analyzed for activation and costimulatory cell surface molecules (Figure 5). Significantly higher percent of activation molecule CD69 (89%; p < 0.05) and costimulatory molecules CD80 (92%; p < 0.05) and CD86 (83%; p < 0.01) were expressed on expanded cells, in presence of both cytokines (IL-2 and IL-15) as compared with IL-2 alone or IL-15 alone. However, no significant differences were observed for HLA-DR under the three different conditions.

### Scheme of γδ T cells expansion method:

**Figure 6:**
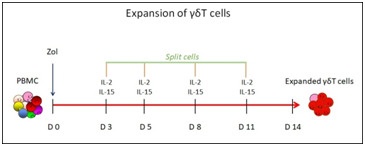
In vitro expansion of human γδ-T cells for 14 days. Concentrations of Zoledronate (Zol) 1 µM. Concentrations of cytokines IL-2 (100 IU/ml) and IL-15 (10 ng/ml). Cells were splits and addition of cytokines and fresh medium, and cells were split on day 3, 5, 8 and 11 of culture.

From these findings, we propose an efficient and reproducible methodology (Figure 6), through which enormous numbers of expanded human γδ T cells can be grown in vitro from a small initial amount of venous blood. This protocol is suitable for most PBMCs obtained from different donors. However, there is a possibility of huge differences in expansion folds, which may vary from donor to donor. In such cases researchers must exercise caution at the beginning of the protocol and should observe and analyze changes accordingly at day 3 - 5.

## Discussion:

γδ T cells are unique unconventional T cells, which are distinguishable from αβ T cells. γδ T cells fulfil numerous important functions in immunity, including cytokine production in response to microbial challenges, mobilization of other types of immune cells and tumor cell killing (at least in vitro) [10, 12,13]. Human blood derived γδ T cells are identified by their unique selectivity for phosphoantigens, such as isopentenyl pyrophosphate (IPP) generated in "stressed" cells and the 10,000-fold more active (E)-4-hydroxy-3-methyl-but-2-enyl pyrophosphate produced by many microbes [13], as well as bisphosphonates such as zoledronic acid [10]. Numerous clinical studies were conducted using γδ T cells as effector cells in cancer patient therapeutics as given in Table 3 (check with authors), including leukemia [14], colorectal carcinoma [15] and renal cell carcinoma, melanoma and acute myeloid leukemia [16]. In this study we have established an in vitro expansion strategy for γ δ T cells, which is an important tool for cancer immune therapeutics. First, PBMCs were isolated from peripheral blood and γδ T cells were expanded in vitro using zoledronate as a stimulant. Standardization of the procedure for washing of PBMCs, suggested that after the first centrifuge of blood with lymphoprepTM, which was at 1680 rpm, all the subsequent washing steps should be done at 1100 rpm for 10 min for better recovery of PBMCs from fresh blood. Extent of in vitro expansion of γδ T cells depends on cytokines. IL-2 is a well-known cytokine, which is secreted by activated cells, and supports further growth of T cells [17]. It is also key for effector T cells development. IL-2 is important especially for antigen guided clonal expansion of T cells [17]. IL-15 is another cytokine which is crucial in this study. Under conditions of infection, naive antigen-specific CD8+ T cells proliferate and further differentiate into CD8+ T lymphocytes [18], which may cause activation-induced cell death, hence limiting the viability and even expansion of cultured T cells [19]. Under such circumstances, IL-15 helps establishing a state of homeostasis of a group of lymphocytes and stimulates T-cell proliferation and survival [20]. Hence, both IL-2 and IL-15 were found to be necessary for the expansion of γδ T cells for 14 days, and significant number of live cells with high percentage of γδ T cells were recovered at 100 IU/ml IL-2 and 10 ng/ml of IL-15. Administration of the bisphosphonates drugs which contain nitrogen (such as zoledronate), in patients of osteoporosis and hypercalcemia in malignancies, lead to enhanced intracellular IPP levels via inhibition of one of the mevalonate pathway enzymes i.e., farnesyl diphosphate synthase. Increase in IPP concentration can lead to activation and expansion of human γδ T cells [21,22]. A stimulant of γδ T cells, known as zoledronate, was also standardized for different concentrations used for the expansion of these cells. One µM concentration of zoledronate exhibited potent stimulation of these cells, well enough to achieve significant percent of γδ T cells population of notable high purity. Relatively high expansion folds were achieved in 14-day cultures, when γδ T cells were stimulated by 1 µM zoledronate with the addition of cytokines IL-2 and IL-15. However, low expansion folds were calculated for expanded γδ T cell with cytokine IL-2 and IL-15 alone. It is evident that combination of both IL-2 and IL-15 considerably increased the activation (CD69) and costimulatory (CD80 and CD86) molecules on expanded γδ T cells cell surface. MHC-class II molecules were expressed on most of the expanded γδ T cells irrespective of cytokine(s) conditions, is a well-known APC marker also T cell activation marker [23,24]. These results were supported by previous finding [22], where γδ T cell cultures were added with IL-15, resulted in observations of a higher proliferative capacity. The expansion folds calculated in this study showed better results than the expansion fold calculated in our previous study [10]. The difference may be due to the use of frozen PBMCs in our previous study. Hence, it is recommended to use fresh blood instead of frozen PBMCs for optimum expansion. We have proposed a scheme based on the findings in this study, which showed an efficient and reproducible method, which may be used to achieve high yield of expanded human γδ T cells using a small amount of blood. These expanded cells can be potentially infused back into the cancer patients or may be frozen down in liquid nitrogen for future use. It was also observed that freshly expanded γδ T cells or even frozen γδ T cells have in vitro tumor cell killing functionality and can also secrete pro-inflammatory cytokines interferon-γ and tumor necrosis factor-α (data not shown) [10, 25]. This research can be extended with the concomitant addition of small biologically active molecules with expanded γδ T cells, which can be tested in vitro as combinational therapeutics. Immune-check point inhibitors used in tumor cytotoxicity assay may also show positive effects in combinational personalized cancer immunotherapies for various tumors.

## Conclusion

It is well established that human γδ T cells have anti-cancer activity and can be safely infused into patients with different cancers. For potential cancer immunotherapy, γδ T cells are needed in large numbers, as several doses of these cells are often required for treatment. Also, it is not feasible to draw large amounts of blood from terminally ill cancer patients, which is the usual procedure for dendritic cell-based immunotherapies. Thus, a standardized method for the expansion of γδ T cells is needed with detailed methodology for their better yield. We have established in this study a detailed method for the expansion of γδ T cells, in which significantly high yields of pure γ δ T cells were recovered with high percentage of live cells after 14 days of culture. Furthermore, these expanded γδ T cells have the potential to be use as personalized cancer therapeutics.

## Figures and Tables

**Table 1:** Isolation of PBMCs from fresh blood at constant time (10 minutes) for different revolutions per minute.

RPM	After 1st Wash		After 2nd Wash		Recovered Live PBMCs
	Cell count	Percent Live Cells	Cell count	Percent Live Cells	
1400	1.71± 0.05 x 106	65%	1.51± 0.02 x 106	61%	0.92± 0.03 x 106
1300	1.63± 0.03 x 106	69%	1.44± 0.03 x 106	67%	0.96± 0.03 x 106
1100*	1.57± 0.04 x 106	79%	1.37± 0.05 x 106	80%	1.09± 0.05 x 106
900	1.52± 0.02 x 106	63%	0.98± 0.04 x 106	63%	0.62± 0.04 x 106
800	1.23± 0.03 x 106	77%	0.74± 0.03 x 106	79%	0.58± 0.04 x 106
600	1.01± 0.03 x 106	76%	0.80± 0.02 x 106	74%	0.59± 0.03 x 106
Cell count before washing started was 2 x 106 in each tube. RPM represents revolutions per minute. Significance is defined as *P< 0.05, when compared between rpm 1100 and 1400, 1300, 900, 800 and 600.

**Table 2:** In vitro expansion of γδ T cells from freshly isolated PBMCs in response to zoledronate (1 µM).

	PBMC + IL-2	PBMC + IL-15	PBMC + IL-2 and IL-15
No. of donors (n)	5	5	5
Day 0			
PBMCs (106/ml)	1	1	1
Start with γδ-T cells (106/ml)	0.017 ± 0.007	0.017 ± 0.007	0.017 ± 0.007
Post Expansion (after 14 days)			
Total Live Cells (%)	69.2 ± 5.3	77.2 ± 7.1	87.5 ± 5.5
CD3+ cells (%)	82.6 ± 7.5	79.6 ± 5.4	95.9 ± 2.7
γδ-T cells (%)	79.4 ± 6.9	84.1 ± 8.2	91.6 ± 5.1
Total cells (106)	11.8 ± 0.9	13.3 ± 0.8	55.8 ± 3.9
*Total γδ-T cells (106)	5.4 ± 1.3	6.8 ± 2.2	38.8 ± 7.1
#Expansion fold	318 ± 121	400 ± 134	2524 ± 787**
Cytokine concentrations IL-2 (100 IU/ml) and IL-15 (10 ng/ml). Each donor PBMCs were cultured in triplicates. Each value is the mean ± SD from 5 different donors *Total γδ-T cells (day 14) were calculated as: total cells x total live cells (%) x CD3+ cells (%) x γδ-T cells (%). #Expansion folds were calculated as: total γδ T cells (day 14) / γδ-T (day 0) **Significance is defined as P < 0.0001 when compared between the expansion fold with the combination of IL2 and IL-15 and IL-2 alone, and between combination of IL-2 and IL-15 and IL-15 alone.
